# Supplementary material for: Health, social, and economic characteristics of patients enrolled in a COVID-19 recovery program
Source: PLoS One. 2022 Nov 30;17(11):e0278154. doi: 10.1371/journal.pone.0278154 (PMC9710845; doi:10.1371/journal.pone.0278154)
Supplement: S1 File — (DOCX) [file pone.0278154.s001.docx]

**Supporting Information: “**Health, Social, and Economic Characteristics of Patients Enrolled in a COVID-19 Recovery Program**”**

|  | **Table of Contents** | **Page(s)** |
| --- | --- | --- |
| S1 Text | PROMIS® -29 Profile v2.1 | 2-4 |
| S2 Text | Fatigue Severity Scale (FSS) | 5 |
| S3 Text | Work Productivity and Activity Impairment Questionnaire: General Health V2.0 (WPAI:GH) | 6-7 |
| S4 Text | Social Determinants of Health Survey (SDOH) | 8 |
| S1 Table | Subgroup Analysis by domain of PROMIS-29 by demographics | 9 |
| S2 Table | Subgroup Analysis by domain of PROMIS-29 by medical history | 10 |
| S3 Table | Subgroup Analysis by domain of PROMIS-29 by demographics for non-normalized distributions | 11 |
| S4 Table | Subgroup analysis by domain of PROMIS-29 by medical history for non-normalized distributions | 12 |
| S1 Checklist | STROBE Statement for cross-sectional studies | 13 |

**PROMIS^®^–29 Profile v2.1**

Please respond to each question or statement by marking one box per row.

| **Physical Function** | | **Without any**  **difficulty** | **With a little**  **difficulty** | **With some**  **difficulty** | **With much**  **difficulty** | **Unable to do** |
| --- | --- | --- | --- | --- | --- | --- |
| PFA11 | Are you able to do chores such as vacuuming or yard work? ......................... | 🞎  5 | 🞎  4 | 🞎  3 | 🞎  2 | 🞎  1 |
|  |  | | | | | |
| PFA21 | Are you able to go up and down stairs  at a normal pace? ...................................... | 🞎  5 | 🞎  4 | 🞎  3 | 🞎  2 | 🞎  1 |
|  |  | | | | | |
| PFA23 | Are you able to go for a walk of at least  15 minutes?............................................... | 🞎  5 | 🞎  4 | 🞎  3 | 🞎  2 | 🞎  1 |
|  |  | | | | | |
| PFA53 | Are you able to run errands and shop? ..... | 🞎  5 | 🞎  4 | 🞎  3 | 🞎  2 | 🞎  1 |
|  | **Anxiety**  **In the past 7 days…** | **Never** | **Rarely** | **Sometimes** | **Often** | **Always** |
| EDANX01 | I felt fearful ............................................... | 🞎  1 | 🞎  2 | 🞎  3 | 🞎  4 | 🞎  5 |
|  |  | | | | | |
| EDANX40 | I found it hard to focus on anything  other than my anxiety ............................... | 🞎  1 | 🞎  2 | 🞎  3 | 🞎  4 | 🞎  5 |
|  |  | | | | | |
| EDANX41 | My worries overwhelmed me ................... | 🞎  1 | 🞎  2 | 🞎  3 | 🞎  4 | 🞎  5 |
|  |  | | | | | |
| EDANX53 | I felt uneasy .............................................. | 🞎  1 | 🞎  2 | 🞎  3 | 🞎  4 | 🞎  5 |
|  | **Depression**  **In the past 7 days...** | **Never** | **Rarely** | **Sometimes** | **Often** | **Always** |
| EDDEP04 | I felt worthless .......................................... | 🞎  1 | 🞎  2 | 🞎  3 | 🞎  4 | 🞎  5 |
|  |  | | | | | |
| EDDEP06 | I felt helpless............................................. | 🞎  1 | 🞎  2 | 🞎  3 | 🞎  4 | 🞎  5 |
|  |  | | | | | |
| EDDEP29 | I felt depressed.......................................... | 🞎  1 | 🞎  2 | 🞎  3 | 🞎  4 | 🞎  5 |
|  |  | | | | | |
| EDDEP41 | I felt hopeless............................................ | 🞎  1 | 🞎  2 | 🞎  3 | 🞎  4 | 🞎  5 |
|  | **Fatigue**  **During the past 7 days…** | **Not at all** | **A little bit** | **Somewhat** | **Quite a bit** | **Very much** |
| HI7 | I feel fatigued.......................................... | 🞎  1 | 🞎  2 | 🞎  3 | 🞎  4 | 🞎  5 |
|  |  | | | | | |
| AN3 | I have trouble starting things because I am tired ................................................... | 🞎  1 | 🞎  2 | 🞎  3 | 🞎  4 | 🞎  5 |

| **Fatigue**  **In the past 7 days…** | | **Not at all** | **A little bit** | **Somewhat** | **Quite a bit** | **Very much** |
| --- | --- | --- | --- | --- | --- | --- |
| FATEXP41 | How run-down did you feel on average? ....................................... | 🞎  1 | 🞎  2 | 🞎  3 | 🞎  4 | 🞎  5 |
|  |  | | | | | |
| FATEXP40 | How fatigued were you on  average? ....................................... | 🞎  1 | 🞎  2 | 🞎  3 | 🞎  4 | 🞎  5 |
|  | **Sleep Disturbance In the past 7 days…** | **Very poor** | **Poor** | **Fair** | **Good** | **Very good** |
| Sleep109 | My sleep quality was ................... | 🞎  5 | 🞎  4 | 🞎  3 | 🞎  2 | 🞎  1 |
|  | **In the past 7 days…** | **Not at all** | **A little bit** | **Somewhat** | **Quite a bit** | **Very much** |
| Sleep116 | My sleep was refreshing. ............. | 🞎  5 | 🞎  4 | 🞎  3 | 🞎  2 | 🞎  1 |
|  |  | | | | | |
| Sleep20 | I had a problem with my sleep ..... | 🞎  1 | 🞎  2 | 🞎  3 | 🞎  4 | 🞎  5 |
|  |  | | | | | |
| Sleep44 | I had difficulty falling asleep ....... | 🞎  1 | 🞎  2 | 🞎  3 | 🞎  4 | 🞎  5 |
|  | **Ability to Participate in Social Roles and Activities** | |  |  |  |  |
|  |  | **Never** | **Rarely** | **Sometimes** | **Usually** | **Always** |
| SRPPER11_CaPS | I have trouble doing all of my  regular leisure activities with others ............................................ | 🞎  5 | 🞎  4 | 🞎  3 | 🞎  2 | 🞎  1 |
|  |  | | | | | |
| SRPPER18_CaPS | I have trouble doing all of the family activities that I want to do.................................................. | 🞎  5 | 🞎  4 | 🞎  3 | 🞎  2 | 🞎  1 |
|  |  | | | | | |
| SRPPER23_CaPS | I have trouble doing all of my usual work (include work at home) ........................................... | 🞎  5 | 🞎  4 | 🞎  3 | 🞎  2 | 🞎  1 |
|  |  | | | | | |
| SRPPER46_CaPS | I have trouble doing all of the activities with friends that I  want to do..................................... | 🞎  5 | 🞎  4 | 🞎  3 | 🞎  2 | 🞎  1 |
|  | **Pain Interference In the past 7 days…** | **Not at all** | **A little bit** | **Somewhat** | **Quite a bit** | **Very much** |
| PAININ9 | How much did pain interfere with your day to day activities? ... | 🞎  1 | 🞎  2 | 🞎  3 | 🞎  4 | 🞎  5 |
|  |  | | | | | |
| PAININ22 | How much did pain interfere with work around the home?........ | 🞎  1 | 🞎  2 | 🞎  3 | 🞎  4 | 🞎  5 |
|  |  | | | | | |
| PAININ31 | How much did pain interfere with your ability to participate  in social activities? ....................... | 🞎  1 | 🞎  2 | 🞎  3 | 🞎  4 | 🞎  5 |

| **Pain Interference**  **In the past 7 days…** | | **Not at all** | | **A little bit** | | **Somewhat** | | **Quite a bit** | | | **Very much** | |
| --- | --- | --- | --- | --- | --- | --- | --- | --- | --- | --- | --- | --- |
| PAININ34 | How much did pain interfere  with your household chores? ......... | 🞎  1 | |  | 🞎  2 |  | 🞎  3 |  | 🞎  4 |  |  | 🞎  5 |
|  | **Pain Intensity**  **In the past 7 days…** | | | | | | | | | | | |
| Global07 | How would you rate your pain  on average? .................................... | 🞎  0 | 🞎  1 | 🞎  2 | 🞎  3 | 🞎  4 | 🞎  5 | 🞎  6 | 🞎  7 | 🞎  8 | 🞎  9 | 🞎  10 |
|  |  | **No**  **pain** |  |  |  |  |  |  |  |  |  | **Worst pain**  **imaginable** |

07 February 2018

© 2008-2018 PROMIS Health Organization (PHO

# FATIGUE SEVERITY SCALE (FSS)

Date Name

Please circle the number between 1 and 7 which you feel best fits the following statements. This refers to your usual way of life within the last week. 1 indicates “strongly disagree” and 7 indicates “strongly agree.”

| Read and circle a number. | Strongly Disagree → Strongly  Agree | | | | | | |
| --- | --- | --- | --- | --- | --- | --- | --- |
| 1. My motivation is lower when I am  fatigued. | 1 | 2 | 3 | 4 | 5 | 6 | 7 |
| 2. Exercise brings on my fatigue. | 1 | 2 | 3 | 4 | 5 | 6 | 7 |
| 3. I am easily fatigued. | 1 | 2 | 3 | 4 | 5 | 6 | 7 |
| 4. Fatigue interferes with my physical functioning. | 1 | 2 | 3 | 4 | 5 | 6 | 7 |
| 5. Fatigue causes frequent problems for me. | 1 | 2 | 3 | 4 | 5 | 6 | 7 |
| 6. My fatigue prevents sustained physical functioning. | 1 | 2 | 3 | 4 | 5 | 6 | 7 |
| 7. Fatigue interferes with carrying out certain duties and responsibilities. | 1 | 2 | 3 | 4 | 5 | 6 | 7 |
| 8. Fatigue is among my most disabling  symptoms. | 1 | 2 | 3 | 4 | 5 | 6 | 7 |
| 9. Fatigue interferes with my work, family,  or social life. | 1 | 2 | 3 | 4 | 5 | 6 | 7 |

# VISUAL ANALOGUE FATIGUE SCALE (VAFS)

Please mark an “X” on the number line which describes your global fatigue with 0 being worst and 10 being normal.

0 1 2 3 4 5 6 7 8 9 10

**Work Productivity and Activity Impairment Questionnaire:**

**General Health V2.0 (WPAI:GH)**

The following questions ask about the effect of your health problems on your ability to work and perform regular activities. By health problems we mean any physical or emotional problem or symptom. *Please fill in the blanks or circle a number, as indicated.*

1. Are you currently employed (working for pay)? ____ NO ____ YES

*If NO, check “NO” and skip to question 6.*

The next questions are about the **past seven days**, not including today.

2. During the past seven days, how many hours did you miss from work because of your health problems? *Include hours you missed on sick days, times you went in late, left early, etc., because of your health problems. Do not include time you missed to participate in this study.*

*_____*HOURS

3. During the past seven days, how many hours did you miss from work because of any other reason, such as vacation, holidays, time off to participate in this study?

_____HOURS

4. During the past seven days, how many hours did you actually work?

_____HOURS *(If “0”, skip to question 6.)*

5. During the past seven days, how much did your health problems affect your productivity while you were working?

*Think about days you were limited in the amount or kind of work you could do, days you accomplished less than you would like, or days you could not do your work as carefully as usual. If health problems affected your work only a little, choose a low number. Choose a high number if health problems affected your work a great deal.*

Consider only how much health problems affected
productivity while you were working.

| Health problems had no effect on my work |  |  |  |  |  |  |  |  |  |  |  | Health problems completely prevented me from working |
| --- | --- | --- | --- | --- | --- | --- | --- | --- | --- | --- | --- | --- |
|  | 0 | 1 | 2 | 3 | 4 | 5 | 6 | 7 | 8 | 9 | 10 |  |

CIRCLE A NUMBER

6. During the past seven days, how much did your health problems affect your ability to do your regular daily activities, other than work at a job?

*By regular activities, we mean the usual activities you do, such as work around the house, shopping, childcare, exercising, studying, etc. Think about times you were limited in the amount or kind of activities you could do and times you accomplished less than you would like. If health problems affected your activities only a little, choose a low number. Choose a high number if health problems affected your activities a great deal.*

Consider only how much health problems affected your ability
to do your regular daily activities, other than work at a job.

| Health problems had no effect on my daily activities |  |  |  |  |  |  |  |  |  |  |  | Health problems completely prevented me from doing my daily activities |
| --- | --- | --- | --- | --- | --- | --- | --- | --- | --- | --- | --- | --- |
|  | 0 | 1 | 2 | 3 | 4 | 5 | 6 | 7 | 8 | 9 | 10 |  |

CIRCLE A NUMBER

WPAI:GH V2.0 (US English)

| 1. Was it OFTEN hard for you to pay for the very basics like food, housing, medical care and heating? | 0=no, 1=yes |
| --- | --- |
| 1. Did you worry whether your food would run out before you got money to buy more? | 0=no, 1=yes |
| 1. Did it happen that the food you bought just didn't last and you didn't have money to get more? | 0=no, 1=yes |
| 1. Have you not been able to pay your rent or mortgage, putting you at risk for eviction? | 0=no, 1=yes |
| 1. Have you had to move in with family/friends or had nowhere to live? | 0=no, 1=yes |
| 1. Were you OFTEN unable to get to activities such as work, school, doctor's appointments, etc., because you did not have access to transportation? | 0=no, 1=yes |
| 1. Has it been hard for you to get OR keep a job? | 0=no, 1=yes |
| 1. In the past 12 months, has the electric, gas, oil or water company threatened to shut off services in your home? | 0=no, 1=yes |

Social Determinants of Health Survey

| Supplement Table 1. Subgroup Analysis by domain of PROMIS-29 by demographics | | | | | | | | | | | | |
| --- | --- | --- | --- | --- | --- | --- | --- | --- | --- | --- | --- | --- |
|  | Age (years) | | | Race/Ethnicity | | | | | | Gender | | |
|  | <50 | 50+ | *p* | Asian | Black | Hispanic | White | Unknown or other | *p* | Male | Female | *p* |
| n | 147 | 120 |  | 11 | 87 | 8 | 148 | 13 |  | 62 | 205 |  |
| Physical Functioning, *mean (s.d)** | 39.5 (7.2) | 39.0 (7.8) | *0.19* | 39.9 (7.1) | 38.8 (7.7) | 35.8 (3.95) | 39.6 (7.6) | 36.2 (5.0) | *0.49* | 40.3 (8.0) | 38.7 (7.3) | *0.09* |
| Anxiety, *mean (s.d)* | 60.0 (8.8) | 57.1 (10.8) | *0.02* | 60.6 (7.5) | 57.3 (12.1) | 65.4 (6.4) | 58.7 (8.7) | 63.3 (6.1) | *0.10* | 56.7 (7.9) | 59.4 (10.3) | *0.07* |
| Depression, *mean (s.d)* | 53.9 (9.3) | 54.0 (9.97) | *0.92* | 54.4 (9.7) | 52.8 (11.1) | 60.2 (7.9) | 54.1 (8.7) | 55.3 (8.3) | *0.36* | 52.7 (8.3) | 54.4 (9.9) | *0.25* |
| Fatigue *mean, (s.d)** | 60.8 (9.4) | 60.6 (10.1) | *0.93* | 59.5 (13.1) | 58.5 (10.0) | 64.7 (6.7) | 61.7 (9.3) | 62.7 (8.4) | *0.10* | 58.5 (8.4) | 61.4 (10.0) | *0.03* |
| Sleep Disturbance, *mean (s.d)* | 55.9 (8.6) | 55.5 (9.3) | *0.72* | 55.6 (12.5) | 56.4 (9.4) | 60.4 (8.2) | 54.6 (8.2) | 62.2 (7.7) | *0.03* | 54.3 (8.3) | 56.1 (9.0) | *0.17* |
| Ability to Participate in Social Roles and Activities, *mean (s.d)** | 42.6 (8.9) | 41.28 (8.55) | *0.33* | 42.4 (11.4) | 43.3 (9.2) | 35.6 (5.3) | 41.1 (8.6) | 38.9 (4.9) | *0.06* | 43.1 (7.7) | 41.7 (9.1) | *0.17* |
| Pain Interference, *mean (s.d)* | 57.5 (10.5) | 56.5 (10.1) | *0.50* | 58.3 (7.6) | 57.4 (11.3) | 60.3 (11.1) | 56.2 (9.9) | 62.6 (9.9) | *0.29* | 55.7 (9.2) | 57.5 (10.7) | *0.27* |
| Pain Intensity, *mean (s.d)* | 4.2 (2.8) | 4.2 (2.8) | *0.93* | 4.6 (3.1) | 4.6 (3.1) | 5.0 (2.6) | 3.9 (2.7) | 5.3 (2.7) | *0.22* | 3.5 (2.4) | 4.4 (2.9) | *0.03* |
| *Values were non-normally distributed values using the D’Agostino-Pearson test and median scores and interquartile ranges are reported in Supplement Table 3 & 4. | | | | | | | | | | | | |

| Supplement Table 2. Subgroup Analysis by domain of PROMIS-29 by medical history | | | | | | | | | | | | |
| --- | --- | --- | --- | --- | --- | --- | --- | --- | --- | --- | --- | --- |
|  | Asthma | | | Allergies | | | Diabetes | | | Hospitalized for COVID-19 | | |
|  | No | Yes | *p* | No | Yes | *p* | No | Yes | *p* | No | Yes | *p* |
| n | 208 | 58 |  | 162 | 104 |  | 242 | 24 |  | 186 | 62 |  |
| Physical Functioning, *mean (s.d)** | 39.6 (7.7) | 37.5 (6.4) | *0.12* | 38.8 (8.0) | 39.5 (6.7) | *0.54* | 39.4 (7.5) | 36.4 (7.0) | *0.061* | 39.7 (7.6) | 37.2 (6.8) | *0.01* |
| Anxiety, *mean (s.d)* | 58.6 (9.9) | 59.1 (9.5) | *0.75* | 59.1 (10.5) | 58.2 (8.9) | *0.49* | 58.8 (9.8) | 58.5 (10.3) | *0.89* | 58.9 (9.5) | 58.4 (10.8) | *0.77* |
| Depression, *mean (s.d)* | 54.0 (9.9) | 53.9 (8.6) | *0.96* | 54.7 (9.9) | 53.0 (9.0) | *0.19* | 53.9 (9.6) | 54.4 (9.3) | *0.81* | 53.9 (9.3) | 54.2 (10.3) | *0.80* |
| Fatigue *mean (s.d)** | 59.9 (9.9) | 63.3 (8.4) | *0.01* | 60.3 (9.9) | 61.2 (9.4) | *0.59* | 60.5 (9.7) | 62.3 (9.4) | *0.52* | 60.7 (9.7) | 60.7 (9.7) | *0.99* |
| Sleep Disturbance, *mean (s.d)* | 55.3 (9.1) | 57.0 (8.3) | *0.22* | 55.6 (8.8) | 55.8 (9.0) | *0.89* | 55.6 (9.0) | 56.3 (8.5) | *0.74* | 55.4 (8.5) | 56.6 (9.9) | *0.39* |
| Ability to Participate in Social Roles and Activities, *mean (s.d)** | 42.8 (8.9) | 39.8 (7.7) | *0.02* | 42.2 (9.1) | 41.9 (8.4) | *0.59* | 42.2 (8.8) | 40.7 (8.8) | *0.633* | 42.4 (8.99) | 41.1 (8.1) | *0.45* |
| Pain Interference, *mean (s.d)* | 56.5 (10.4) | 59.0 (9.8) | *0.10* | 56.7 (11.0) | 57.6 (9.4) | *0.49* | 56.9 (10.3) | 58.3 (10.5) | *0.55* | 57.3 (10.0) | 56.4 (11.4) | *0.59* |
| Pain Intensity, *mean (s.d)* | 4.1 (2.9) | 4.8 (2.5) | *0.08* | 4.0 (3.0) | 4.5 (2.5) | *0.23* | 4.1 (2.8) | 5.0 (3.0) | *0.13* | 4.2 (2.6) | 4.2 (3.0) | *0.99* |
| *Values were non-normally distributed values using the D’Agostino-Pearson test and median scores and interquartile ranges are reported in Supplement Table 3 & 4. | | | | | | | | | | | | |

| Supplement Table 3. Subgroup Analysis by domain of PROMIS-29 by demographics for non-normalized distributions | | | | | | | | | | | | |
| --- | --- | --- | --- | --- | --- | --- | --- | --- | --- | --- | --- | --- |
|  | Age (years) | | | Race/Ethnicity | | | | | | Gender | | |
|  | <50 | 50+ | *p* | Asian | Black | Hispanic | White | Unknown or other | *p* | Male | Female | *p* |
| n | 147 | 120 |  | 11 | 87 | 8 | 148 | 13 |  | 62 | 205 |  |
| Physical Functioning, *median [interquartile range]* | 37.90 [35.60, 43.40] | 36.70 [33.30, 41.80] | *0.19* | 39.10 [35.00, 42.60] | 36.70 [34.12, 41.80] | 35.60 [33.85, 36.70] | 36.70 [35.00, 39.15] | 37.90 [34.40, 43.40] | *0.49* | 39.10 [36.70, 45.30] | 36.70 [34.40, 41.80] | *0.09* |
| Fatigue, *median [interquartile range]* | 62.70 [56.52, 66.70] | 60.70 [54.60, 66.70] | *0.93* | 64.60 [50.05, 69.00] | 58.80 [51.00, 64.60] | 62.70 [58.80, 69.00] | 64.60 [59.85, 67.85] | 62.70 [57.00, 66.70] | *0.10* | 58.80 [53.10, 64.60] | 62.70 [57.00, 66.70] | *0.03* |
| Ability to Participate in Social Roles and Activities, *median [interquartile range]* | 42.30 [37.30, 48.10] | 40.50 [35.70, 46.20] | *0.33* | 37.30 [35.70, 50.95] | 44.20 [37.30, 48.10] | 35.70 [32.90, 38.05] | 37.30 [35.65, 40.50] | 42.30 [35.70, 45.20] | *0.06* | 44.20 [37.30, 48.10] | 40.50 [35.70, 46.20] | *0.17* |

| Supplement Table 4. Subgroup analysis by domain of PROMIS-29 by medical history for non-normalized distributions | | | | | | | | | | | | |
| --- | --- | --- | --- | --- | --- | --- | --- | --- | --- | --- | --- | --- |
|  | Asthma | | | Allergies | | | Diabetes | | | Hospitalized for COVID-19 | | |
|  | No | Yes | *p* | No | Yes | *p* | No | Yes | *p* | No | Yes | *p* |
| n | 208 | 58 |  | 162 | 104 |  | 242 | 24 |  | 186 | 62 |  |
| Physical Functioning, *median [interquartile range]* | 37.90 [34.40, 43.40] | 36.70 [33.57, 0.40] | *0.12* | 36.70 [34.40, 42.20] | 37.90 [34.40, 42.20] | *0.54* | 37.90 [34.40, 43.40] | 36.70 [32.10, 39.10] | *0.06* | 39.10 [34.40, 43.40] | 36.70 [33.30, 40.40] | *0.01* |
| Fatigue, *median [interquartile range]* | 60.70 [53.10, 66.70] | 64.60 [58.80, 9.00] | *0.01* | 62.70 [54.60, 66.70] | 62.70 [57.00, 66.70] | *0.59* | 62.70 [55.10, 66.70] | 62.70 [58.35, 67.93] | *0.52* | 62.70 [55.10, 66.70] | 60.70 [57.00, 66.70] | *0.99* |
| Ability to Participate in Social Roles and Activities, *median [interquartile range]* | 42.30 [37.30, 48.10] | 38.80 [36.10, 4.20] | *0.02* | 40.50 [37.30, 48.10] | 42.30 [37.30, 45.20] | *0.59* | 40.50 [37.30, 47.15] | 41.40 [33.45, 48.58] | *0.63* | 42.30 [37.30, 48.10] | 40.50 [36.90, 46.20] | *0.45* |

| Table 5. STROBE Statement for cross-sectional studies | | |  |
| --- | --- | --- | --- |
|  | Item No | Recommendation | Page No |
| **Title and abstract** | 1 | (*a*) Indicate the study’s design with a commonly used term in the title or the abstract | 3 |
|  |  | (*b*) Provide in the abstract an informative and balanced summary of what was done and what was found | 3 |
| Introduction | | | |
| Background/rationale | 2 | Explain the scientific background and rationale for the investigation being reported | 5-6 |
| Objectives | 3 | State specific objectives, including any prespecified hypotheses | 6 |
| Methods | | | |
| Study design | 4 | Present key elements of study design early in the paper | 5-6 |
| Setting | 5 | Describe the setting, locations, and relevant dates, including periods of recruitment, exposure, follow-up, and data collection | 6-7 |
| Participants | 6 | (*a*) Give the eligibility criteria, and the sources and methods of selection of participants | 7 |
| Variables | 7 | Clearly define all outcomes, exposures, predictors, potential confounders, and effect modifiers. Give diagnostic criteria, if applicable | 7-9 |
| Data sources/ measurement | 8* | For each variable of interest, give sources of data and details of methods of assessment (measurement). Describe comparability of assessment methods if there is more than one group | 7-9 |
| Bias | 9 | Describe any efforts to address potential sources of bias | 6-7 |
| Study size | 10 | Explain how the study size was arrived at | 9 |
| Quantitative variables | 11 | Explain how quantitative variables were handled in the analyses. If applicable, describe which groupings were chosen and why | 9 |
| Statistical methods | 12 | (*a*) Describe all statistical methods, including those used to control for confounding | 9 |
|  |  | (*b*) Describe any methods used to examine subgroups and interactions | 9 |
|  |  | (*c*) Explain how missing data were addressed | 9 |
|  |  | (*d*) If applicable, describe analytical methods taking account of sampling strategy |  |
|  |  | (*e*) Describe any sensitivity analyses |  |
| Results | | | |
| Participants | 13* | (a) Report numbers of individuals at each stage of study—eg numbers potentially eligible, examined for eligibility, confirmed eligible, included in the study, completing follow-up, and analysed | 10-12 |
|  |  | (b) Give reasons for non-participation at each stage |  |
|  |  | (c) Consider use of a flow diagram |  |
| Descriptive data | 14* | (a) Give characteristics of study participants (eg demographic, clinical, social) and information on exposures and potential confounders | 10-12 |
|  |  | (b) Indicate number of participants with missing data for each variable of interest |  |
| Outcome data | 15* | Report numbers of outcome events or summary measures | 13-17 |
| Main results | 16 | (*a*) Give unadjusted estimates and, if applicable, confounder-adjusted estimates and their precision (eg, 95% confidence interval). Make clear which confounders were adjusted for and why they were included | 13-17 |
|  |  | (*b*) Report category boundaries when continuous variables were categorized |  |
|  |  | (*c*) If relevant, consider translating estimates of relative risk into absolute risk for a meaningful time period |  |
| Other analyses | 17 | Report other analyses done—eg analyses of subgroups and interactions, and sensitivity analyses | 17 |
| Discussion | | | |
| Key results | 18 | Summarise key results with reference to study objectives | 18 |
| Limitations | 19 | Discuss limitations of the study, taking into account sources of potential bias or imprecision. Discuss both direction and magnitude of any potential bias | 19-20 |
| Interpretation | 20 | Give a cautious overall interpretation of results considering objectives, limitations, multiplicity of analyses, results from similar studies, and other relevant evidence | 19-20 |
| Generalisability | 21 | Discuss the generalisability (external validity) of the study results | 18-20 |
| Other information | | | |
| Funding | 22 | Give the source of funding and the role of the funders for the present study and, if applicable, for the original study on which the present article is based | 2 |

*Give information separately for exposed and unexposed groups.

**Note:** An Explanation and Elaboration article discusses each checklist item and gives methodological background and published examples of transparent reporting. The STROBE checklist is best used in conjunction with this article (freely available on the Web sites of PLoS Medicine at http://www.plosmedicine.org/, Annals of Internal Medicine at http://www.annals.org/, and Epidemiology at http://www.epidem.com/). Information on the STROBE Initiative is available at www.strobe-statement.org.
